# The Roles of Exosomes upon Metallic Ions Stimulation in Bone Regeneration

**DOI:** 10.3390/jfb13030126

**Published:** 2022-08-24

**Authors:** Xuwei Luo, Dongqin Xiao, Chengdong Zhang, Guanglin Wang

**Affiliations:** 1 Orthopedic Research Institute, Department of Orthopedics, West China Hospital, Sichuan University, Chengdu 610041, China; 2 Research Institute of Tissue Engineering and Stem Cells, Nanchong Central Hospital, The Second Clinical College of North Sichuan Medical College, Nanchong 637000, China

**Keywords:** metallic ions, exosomes, bone substitutes, bone regeneration

## Abstract

Metallic ions have been widely investigated and incorporated into bone substitutes for bone regeneration owing to their superior capacity to induce angiogenesis and osteogenesis. Exosomes are key paracrine mediators that play a crucial role in cell-to-cell communication. However, the role of exosomes in metallic ion-induced bone formation and their underlying mechanisms remain unclear. Thus, this review systematically analyzes the effects of metallic ions and metallic ion-incorporated biomaterials on exosome secretion from mesenchymal stem cells (MSCs) and macrophages, as well as the effects of secreted exosomes on inflammation, angiogenesis, and osteogenesis. In addition, possible signaling pathways involved in metallic ion-mediated exosomes, followed by bone regeneration, are discussed. Despite limited investigation, metallic ions have been confirmed to regulate exosome production and function, affecting immune response, angiogenesis, and osteogenesis. Although the underlying mechanism is not yet clear, these insights enrich our understanding of the mechanisms of the metallic ion-induced microenvironment for bone regeneration, benefiting the design of metallic ion-incorporated implants.

## 1. Introduction

### 1.1. Metallic Ions and Bone Healing

In recent decades, with rapid economic advancement and the aging population, large bone defects caused by musculoskeletal diseases (e.g., osteoporosis, trauma, bone tumors, and spinal disorders) have affected hundreds of millions of people across the world. Autografts from patients are considered the gold standard for clinical treatment. However, limited donor resources and complications have restricted its widespread application. Presently, orthopedic implants derived from biomaterials are used in therapeutic strategies, such as calcium phosphate as a bone defect filler [1] or titanium alloys as intervertebral cages for spine fusion [2]. Although many bone substitutions are available, inferior healing effects (e.g., poor vascularization and bone bonding) have led researchers to explore more osteoinductive biomaterials that are similar to natural bone.

**Figure 1:**
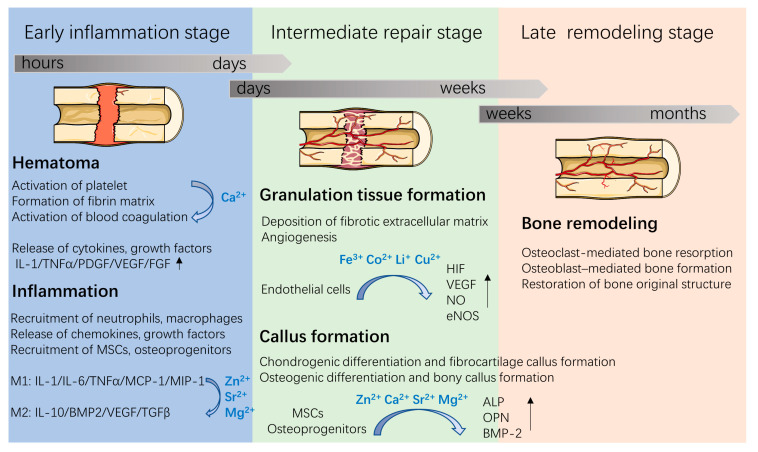
Three overlapping stages involved in the bone healing process and roles of metallic ions during bone repair. Figure was produced using Servier Medical Art (http://smart.servier.com/ (accessed on 5 January 2022)).

As a part of the musculoskeletal system, bone plays a role in both the support and movement of the body, and is a storage reservoir for calcium, phosphorus, and other trace elements. Bone is mainly composed of inorganic minerals (50–70% calcium phosphate), collagen fibers (20–40%), and water (5–10%). The inorganic minerals in bones are mainly carbo-hydroxyapatite (HA); meanwhile, trace elemental substitutions exist in biological apatite. For example, metallic ions (such as Mg^2+^, Zn^2+^, Sr^2+^) and anions (such as CO_3_^2−^, SiO_3_^2−^, F^−^) are co-incorporated in the apatite structure. Various metallic elements have been proven to play key roles in bone formation and healing processes [3,4,5] (Figure 1). For example, Sr^2+^, Mg^2+^, and Ca^2+^ can enhance osteogenesis, while Cu^2+^, Co^2+^, Li^+^, and Fe^3+^ can increase neovascularization. Specifically, approximately 99% of Ca is stored in bone in forms of HA. Ca acts as an ionic messenger and participates in a series of cellular process, including exocytosis, apoptosis, and motility. During bone healing processes, Ca^2+^ plays key roles in the activation and aggregation of platelets, blood clot formation, the stimulation of bone mineralization, and subsequent bone healing [3]. Moreover, approximately 50% of Cu and Mg are stored in the musculoskeletal system while 90% of Sr exists in bone tissue. Although these trace metallic ions are minor components in bone tissues, they act as enzymes cofactors and participate in bone metabolism and remodeling. Sr ions enhance immune suppression and promote osteogenesis [4]. Fe ions regulate various cells (e.g., mesenchymal stem cells (MSCs) and endothelial cells) functions and promote angiogenesis by increasing hypoxia-inducible factor-1α (HIF-1α) levels, vascular endothelial growth factor (VEGF) expression and endothelial NO synthase (eNOS) production [5,6]. Mg ions induce an anti-inflammatory environment and enhance osteogenic differentiation by activating the bone morphogenetic protein 2 (BMP-2) signaling pathway in MSCs [7].

With the evolving understanding of bone remodeling, it has been realized that the bone healing process involves an orchestrated series of biological events (Figure 1). First, after implantation, proteins in the blood and in tissue fluids are immediately adsorbed on the implant surface, a process that is followed by hematoma formation, which is regulated by plasma proteases. These are responsible for fibrin formation and platelet activation [8]. The formed hematomas, as a fibrin scaffold, support cell adhesion and release chemokines, proinflammatory cytokines, and growth factors (e.g., platelet-derived growth factor (PDGF), VEGF, fibroblast growth factor (FGF), and tumor necrosis factor-α (TNFα)) to recruit and activate neutrophils and macrophages. Then, the inflammatory cells release cytokines and recruit MSCs and osteoprogenitors, thus stimulating angiogenesis, extracellular matrix synthesis and bone remodeling at the injury site. Since metallic ions are involved in the above bone healing process, incorporating metallic ions into bone implants has attracted great attention in bone tissue engineering.

### 1.2. Exosomes and Bone Healing

In bone tissue regeneration, scaffolds both offer mechanical support for cell adhesion, and provide a micro-environment comprising surface structure, chemical composition, and mechanical signal, which regulates cell behavior and tissue healing. The micro-environment provided by the implants has been proven to be a key modulator of cell–material interactions and cell-to-cell communications by stimulating or inhibiting cell-associated signals. Various cells, including MSCs, endothelial cells, osteoblasts, osteoclasts, and immune cells, are involved in the biological healing process around the implant surface. The communications among various cells are vital to the cell functions and tissue regeneration. In addition to direct cell–cell contact, paracrine communication also occurs among cells located far apart from each other via secreted factors. Among the various secreted factors, here, we will focus on the roles of exosomes in cell-to-cell communication and bone regeneration.

As a kind of cell vesicles, exosomes were first discovered in sheep reticulocytes in 1983. Exosomes are extracellular vesicles with diameter of 30–150 nm, which are secreted by a variety of cells (e.g., MSCs, adipocytes, dendritic cells, and epithelial cells) and present in blood, urine, saliva, and other bodily fluids [9,10]. Exosomes, known as important intercellular communication mediators, play an important role in regulating cell-to-cell communication through delivering selected nuclei acids, lipids, and proteins. Meanwhile, exosomes have showed similar biofunctions with their derived cells and low immune rejection [11,12]. Thus, exosomes have been used to promote the process of bone tissue regeneration, avoiding the safety concerns of direct cell transplantation. For instance, MSC-derived exosomes significantly enhanced the proliferation and osteogenic differentiation of bone marrow mesenchymal stem cells (BMSCs) in distraction osteogenesis-mediated bone regeneration in older rats [13]. Additionally, M2 macrophage-derived exosomes could enhance the osteogenesis and decrease the adipogenesis of BMSCs, exhibiting positive paracrine regulation of bone regeneration [14,15]. Despite these advancements, the clinical application of exosomes is limited by the extraction method and large dose requirements. In addition, the biofunction and production capability of exosomes are relative to various factors, e.g., culture microenvironment and cell types. Therefore, the modification of the cell microenvironment or of cell themselves has potential for achieving exosomes with superior therapeutic effects for regenerative medicine.

It is noteworthy that when biomaterials co-cultured with cells, the various chemical signals derived from implants can directly act on the cells, which is followed by cell behaviors being affected (e.g., through adhesion, proliferation, and differentiation). Additionally, the released chemical signals can change the microenvironment where such cells are located, therefore affecting the exosome secretion from cells. Findings in the literature combined with our previous studies confirmed that metallic ions incorporated with bone substitutes could obviously enhance the angiogenic and osteogenic capacities of various cells and promote new bone formation in vivo [16,17,18]. Due to the fact that exosome production is determined by cell behavior, and exosomes are involved in intercellular paracrine communication, metallic ions released from implants may influence cell behavior by modifying exosome secretion from various cells. Recent research efforts focused on studying the effects of ion signal on exosome secretion and osteogenesis [19]. Therefore, understanding the roles of metallic ions in regulating the exosome secretion of cells is crucial when designing biomaterials for bone regeneration. This review mainly focuses on the roles of metallic ions in the secretion of exosomes from MSCs and macrophages by elaborating on cell–material interactions and bone regeneration.

## 2. Exosomes Derived from Different Cells Relevant to Bone Regeneration

### 2.1. MSCs-Derived Exosomes

MSCs are multipotent stromal cells which exist in connective tissues that can differentiate into multiple cell types (e.g., bone, fat, nerves, etc.) and have strong self-renewing abilities. According to their isolated locations, MSCs are mainly divided into adipose tissue-derived MSCs (ADMSCs), bone marrow-derived MSCs (BMSCs), dental pulp-derived MSCs (DPMSCs), umbilical cord-derived MSCs (UCMSCs) and peripheral blood MSCs (PBMSCs), etc. As the most commonly used stem cells, MSCs have been widely used to improve bone tissue repair, including the local injection of MSCs with/without scaffolds [20,21]. However, problems such as risk, immune response and how to achieve stable MSCs rapidly have always been challenging in clinical applications. Many studies proved that the therapeutic effects of MSCs are attributed not only to their differentiation into bone forming-related cells but mainly to their paracrine function, including secreting growth factors, cytokines, and chemokines, and releasing extracellular vesicles such as exosomes [22,23]. Exosomes secreted by MSCs display similar biological functions as those of their parental cells. Therefore, a new therapeutic strategy based on MSCs-derived exosomes has been applied in bone tissue repair as summarized in Table 1.

MSC-derived exosomes have various regenerative capabilities, including guiding immunomodulatory regulation, angiogenesis, and osteogenesis. Thus, MSC-derived exosomes have potential for treating bone diseases, such as osteoarthritis, osteoporosis, and bone fractures, using their biological cargoes (e.g., proteins and miRNAs). Compared with MSC-based therapies in bone regeneration, MSC-derived exosomes show some advantages, such as non-living, stable preservation in the body and high loading capacity for RNAs and proteins. Moreover, exosomes derived from different MSCs exhibit various therapeutic effects. Wang et al. reported that ADMSC-derived exosomes have superior immune regulation capacity, BMSC-derived exosomes have excellent regeneration ability, and UCMSC-derived exosomes play a vital role in tissue damage repair [24]. In addition, another study indicated that DPMSC-derived exosomes show more prominent immune-modulating ability than BMSC-derived exosomes [25]. The different therapeutic effects are highly relevant to the cargoes in the exosomes, including microRNAs and proteins (see Table 1). However, whether these differences have effects, and the question of how these differences can be triggered, require further study.

**Table 1:** Exosomes derived from various MSCs and their functions in bone regeneration.

Sources	Markers	Cargoes	Functions	Ref.
ADMSCs-derived exosomes	CD9, CD63	miR-375 overexpression	Enhance the osteogenic differentiation of BMSCs by inhibiting IGFBP3 proteins via miR-375 overexpression in exosomes;	[26]
	CD9, CD63, Tsg101, CD81	miR-34, miR-146, miR-21 upregulation	Shift macrophages from M1 to M2 phenotype using exosomes from ADMSCs pr-activated with inflammatory cytokines (IFNγ/TNFα) via miRNA regulation	[27]
BMSCs-derived exosomes	CD9, CD63	miR-150-3p upregulation	Attenuate osteoporosis by promoting osteoblast proliferation, differentiation and inhibiting apoptosis via miR-150-3p upregulation in exosomes;	[28]
	CD9, CD63, Hsp70	miR-26a-5p overexpression	Alleviate osteoarthritis by down-regulation of PTGS2 followed by inhibiting synovial fibroblasts proliferation and inflammation via miR-26a-5p overexpression in exosomes;	[29]
	CD63, CD81	miR-128-3p upregulation	Attenuate osteogenesis and bone fracture healing via upregulation of miR-128-3p in aged-exosomes via targeting Smad5 followed by reducing RUNX2, ALP and Col I	[30]
	CD9, CD63, CD81	Undetected	Enhance osteogenesis, angiogenesis and bone healing process by transplantation of exosomes in vivo via activating BMP-2/Smad1/RUNX2 signaling pathway	[31]
UCMSCs-derived exosomes	CD9, CD81, CD63	Undetected	Accelerate fracture healing by implantation of exosome via inducing HIF-α and followed angiogenesis;	[32]
	CD9, CD81, CD63	Undetected	Enhance bone regeneration using exosomes via promoting osteoblast migration and the expression levels of osteogenic genes (ALP, OCN, COL1A1)	[33]
DPMSCs-derived exosomes	CD9, CD63	Undetected	Exhibit strong immune-modulating activity by reducing the secretions of pro-inflammatory factors IL-17, TNF-α and IL-17 as well as increasing the anti-inflammatory factors IL-10 and TGF-β	[25]

### 2.2. Macrophage-Derived Exosomes

Recently, the crosstalk between bone cells and immune cells has become regarded to be a vital factor in tissue homeostasis and new bone formation. Among these cells, the effect of macrophages on bone metabolism has attracted great attention. Macrophages possess different functional states under various pathophysiological conditions that could affect bone repair. Macrophages can be divided into nonactivated (M0), classically activated (M1), and alternatively activated (M2) subtypes. M1 macrophages can induce a pro-inflammatory response and enhance the host’s defense reaction by producing high levels of reactive oxygen species (ROS), nitric oxide (NO), and proinflammatory cytokines (e.g., TNF-a, interleukin 1 (IL-1), IL-2, IL-6, and IL-12). Conversely, M2 macrophages can produce anti-inflammatory cytokines (e.g., IL-10, CCL-18, and CCL-22) and enhance immune suppression. Meanwhile, M2 macrophages can produce growth factors, including VEGF and BMP-2, and can accelerate bone fracture healing [34]. Both M2 and M0 macrophages can enhance the osteogenic differentiation of MSCs [35].

Similarly, macrophages secrete exosomes that are important for cell-to-cell communication, immune regulation, and tissue regeneration. Exosomes derived from M0, M1, and M2 exert different effects in bone healing. Studies proved that M1 macrophage-derived exosomes have inhibitory effects on bone healing, whereas M2 macrophage-derived exosomes elicit osteoinductive effects in bone regeneration [15]. Moreover, Xia et al. proved that exosomes derived from M0, M1, and M2 all suppressed the chondrogenic differentiation of MSCs [36]. Functional variation may be attributed to different substances in exosomes derived from different types of macrophages (Table 2). For instance, miRNA-5106 was found to be overexpressed in M2 macrophage-derived exosomes, but was found to be decreased in M1 macrophage-derived exosomes [37]. Additionally, miRNA-5106-enriched exosomes could enhance the osteogenic differentiation of BMSCs by inhibiting the expression of salt-inducible kinase 2 (SIK2) and SIK3 genes [37], which are involved in cell cycle regulation and differentiation. In addition, Yu et al. [38] found that M1 macrophage-derived exosomes aggravated bone loss by enhancing miRNA-98 expression in osteoblasts, followed by suppressing DUSP1 and activating the JNK signaling pathway. Moreover, macrophage-derived exosomes possess many alarmins, such as annexin, galectin, and fibronectin [39,40], which are endogenous molecules released upon tissue damage and stimulate inflammation. Annexins, as exosomal membrane-associated proteins, can enhance the phagocytic capacity of macrophages, thus accelerating bone resorption [41]. Galectin, as a lectin enriched in exosomes from young individuals, can improve the osteogenic differentiation of MSCs [40], while fibronectin presented on the exosome surface can regulate the binding of integrin-dependent exosomes to target cells [42]. Despite these potential applications of exosomes in bone regeneration, the knowledge about MSC- or macrophage-derived exosomes is limited. Therefore, further studies are required to determine exosome functions and mechanisms.

## 3. Exosome Secretion upon Metallic Ions Stimulation and the Effects on Bone Regeneration

**Figure 2:**
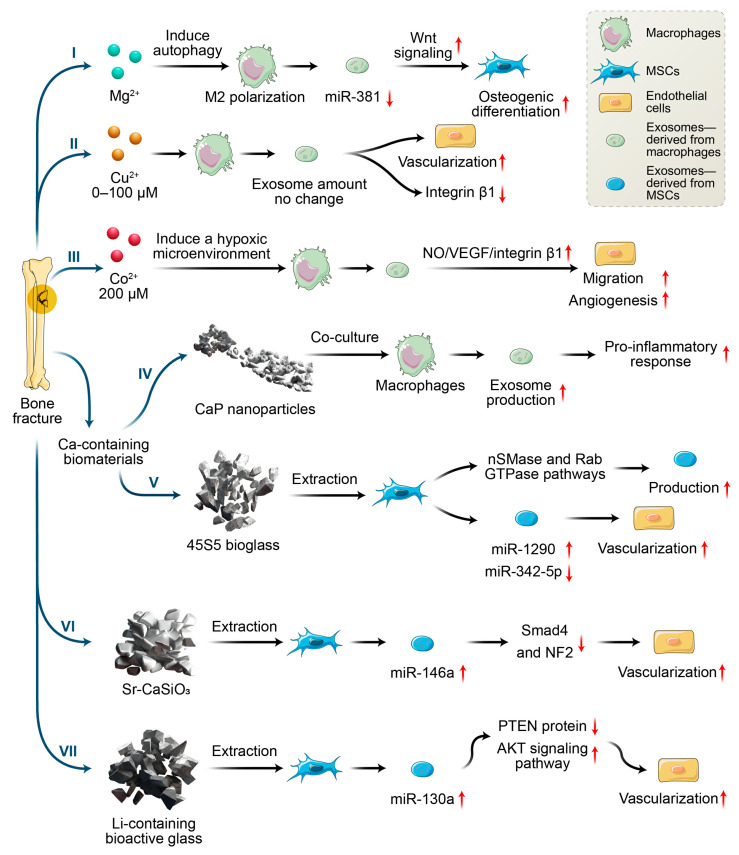
Effects of metallic ions or metallic ions incorporated with biomaterials on angiogenesis and osteogenesis via macrophages and MSCs secreting exosomes.

In recent years, the incorporation of metallic ions into biomaterials has been regarded as a promising strategy for inducing osteogenesis and angiogenesis for bone regeneration. Various approaches to incorporate metallic ions into biomaterials have been explored and have been proven to have positive effects on in vitro and in vivo bone healing [43,44]. When biomaterials are in contact with cells, the biomaterials both directly act on cells and affect cell-to-cell communications by changing the microenvironments of the cells’ locations. For instance, Zn^2+^/Sr^2+^ released from collagen/HA can enhance the communication between macrophages and BMSCs by inducing an osteoimmune microenvironment [45]. Zn^2+^/Sr^2+^ release up-regulated the osteogenic genes (e.g., BMP-2, Wnt10b) of macrophages, and then enhanced the osteogenesis-related gene expression (e.g., OCN, OPN, and ALP) of BMSCs and bone regeneration in vivo. However, the cell-to-cell communication mechanisms involved in the chemical signals derived from biomaterials are complex and are difficult to clarify. In addition to the conventional approaches to investigating mechanisms, exosomes have emerged as potential candidates with paracrine capacity which has been contributed to cell-to-cell communication. In addition, exosomes production and functions are highly dependent on physiological conditions, suggesting that variations in the microenvironment can modify the production of exosomes. However, reports on the effects of metallic ions on exosomes secretion are limited. Nevertheless, recent research efforts focused on this area, and may provide insight for us to design biomaterials for bone regeneration. Here, we mainly focus on the roles of those metallic ions that are widely incorporated into bone substitutes in exosomes production, as well as the biological function in intercellular communication and bone regeneration (Figure 2).

### 3.1. Magnesium Ions

As the fourth most abundant element in the human body, magnesium ions participate in various metabolic activities, such as protein and nuclei acid synthesis, enzymatic reactions, and transmembrane ion transport. Moreover, approximately half of Mg^2+^ is stored in bone tissue, which directly modifies the bone-calcification process and the generation of new minerals in the human body. Mg^2+^ deficiency induces the release of inflammatory factors, resulting in a series of clinical disorders such as migraine, metabolic syndrome, hypertension, and atherosclerosis [46]. Additionally, Mg^2+^ deficiency causes osteoporosis by reducing the activity of osteoblasts and osteoclasts, and inhibiting apatite deposition. In addition, low Mg^2+^ concentration disturbs the phosphorus acid inositol system and/or reduces adenylate cyclase activity, inducing antiparathyroid hormone effects which are followed by decreased bone strength [47]. Conversely, Mg^2+^ addition both enhances the expression of the proteins and genes related to osteogenic differentiation and increases nitric oxide production, which can induce angiogenesis as well as prevent inflammation [48,49].

Despite these findings, current researchers have seldom focused on the influence of Mg^2+^ on bone regeneration from the perspective of exosomes. A study by Zhu et al. [49] was one of the few to study the effects of exosomes derived from Mg^2+^-treated macrophages on osteogenesis. They found that Mg^2+^ addition enhanced the M2 polarization of macrophages and increased TGF-β, as well as decreasing the expression of miR-381 in exosomes derived from macrophages through autophagy. Additionally, exosomes with reduced miR-381 were demonstrated to promote the osteogenic differentiation of BMSCs. Previous studies proved that miR-381 can be regarded as a tumor suppressor in various cancers such as breast cancer and osteosarcoma [50,51]. Additionally, another study indicated that miR-381 overexpression inhibited the osteogenic differentiation of BMSCs by suppressing Wnt signaling, while the down-regulation of miR-381 enhanced bone fracture healing [52]. Thus, down-regulation of exosomal miR-381 derived from macrophage upon exogenous Mg^2+^ stimulation, may provide a new direction for enhancing the osteogenic differentiation of MSCs.

### 3.2. Copper Ions

As the second essential trace element in humans, copper ions serve as a cofactor and vital component for many enzymes, affecting the development and function of the blood, immune system, and bones, etc. More than 50% of copper is distributed in the muscles and bones. Copper ions participate in the metabolism of connective tissue, bones, and epiphyseal cartilage, which promote angiogenesis and inhibit osteoporosis [53]. In addition, copper has good antibacterial properties against both Gram-positive and Gram-negative bacteria [54]. Ryan et al. [55] combined Cu^2+^-eluting bioactive glass into collagen scaffolds and found that the scaffolds incorporated with Cu^2+^ showed antibacterial ability and enhanced angiogenesis and osteogenesis compared with scaffolds without the addition of copper ions. Studies proved that copper ion-induced angiogenesis may be caused by enhancing VEGF secretion and the migration of vascular endothelial cells [56]. Additionally, copper ions can polarize macrophages toward to M1 phenotypes [57,58], and facilitate angiogenesis via VEGF secretion [59]. VEGF secretion may be one of the mechanisms contributing to pro-angiogenesis; however, the effect of copper ion-stimulated exosomes on angiogenesis is still unclear. Wang et al. [58] found that copper ions (concentration: 0–100 µM) have no significant effect on the exosome secretion from macrophages, but the exosomes did reduce the surface integrin β1 of endothelial cells. As the family member of integrins, integrin β1 plays key roles in the adhesion, migration, proliferation, and angiogenesis of endothelial cells. Meanwhile, Tanjore et al. proved that integrin β1 was vital for embryonic angiogenesis but not essential for vasculogenesis. Despite decreased integrin β1, endothelial cells showed enhanced angiogenic capacity when cocultured with the exosomes derived from macrophages upon copper ion stimulation [58]. The results suggested that other factors might promote the angiogenesis of endothelial cells, such as the miRNAs in exosomes. Wang et al. [58] proposed that copper stimulation might up-regulate the pro-angiogenic RNAs and down-regulate anti-angiogenic RNAs in exosomes. Although the mechanism remains to be studied further, the investigation demonstrated that exosomes derived from macrophages upon copper stimulation were pro-angiogenic, which is helpful for the design of copper ion-incorporated bone implants.

### 3.3. Cobalt Ions

As an essential element in the human body, cobalt ions play a key role in biological activities by complexing with various proteins (e.g., vitamin B12). Cobalt ions serve as hypoxia-mimicking agents for promoting tissue angiogenesis by enhancing the secretion of HIFs. Additionally, studies proved that the hypoxia condition promotes osteogenesis at an early stage by inducing an HIF1a–Twist1 pathway while suppresses osteogenesis maturation at the late stage by inhibiting the HIF1a–Twist1 pathway [60]. Thus, the dose-dependent behavior of cobalt ions, implemented at an appropriate time, is essential for mediating oxygen levels and the bone regeneration that follows. Wu et al. [61] fabricated hypoxia-mimicking bioactive glass scaffolds by incorporating cobalt ions into the scaffolds and found that this incorporation enhanced hypoxia functions through increasing HIF-1α expression and VEGF secretion. This promoted BMSC proliferation and osteogenic gene expression. Another study [62] found that the cobalt-induced osteogenic differentiation of BMSCs was eliminated when macrophages were included. The extraction of cobalt ion-incorporated tricalcium phosphate induced macrophages toward a proinflammatory M1 phenotype; this was accompanied by fibrous encapsulation instead of new bone formation in vivo [62]. However, although cobalt ion-incorporated tricalcium phosphate fails in bone repair, we cannot conclude that cobalt-ion-incorporated implants should be abandoned. Instead, findings have indicated the essential roles of macrophages in the evaluation of biomaterial-induced osteogenesis.

Based on the key roles of exosomes in intercellular communication, the underlying mechanism of exosomes derived from macrophages upon cobalt stimulation on angiogenesis were studied by Zhang et al. [63]. They found that the exosomes could enhance endothelial migration and angiogenesis both in vitro and in vivo when cobalt concentration was 200 µM. These results could possibly be attributed to the enhanced release of nitric oxide (NO), VEGF, and integrin β1 from exosome-regulated endothelial cells upon cobalt stimulation. As an endothelium-derived relaxing factor (EDRF), NO can increase VEGF secretion by enhancing HIF-1α activity through the PI3K-Akt signaling pathways [64]. Conversely, VEGF can increase NO production by enhancing eNOS activation through the PKC- and CaM-Akt signaling pathways [64]. These interactions are beneficial for angiogenesis. However, no significant difference in exosomal VEGF content was observed in the study by Zhang et al. [63]. The authors speculated that this might be attributed to the miRNAs in the exosomes, although no further study has yet been carried out. Admittedly, the presence of miRNAs in exosomes has been considered a critical factor in angiogenesis. For example, exosomal miR-210 and miR-135b have been proven to enhance angiogenesis by targeting HIF-1α [65,66]. Notably, miR-210 is a hypoxia-responsive miRNA, which shows high expression in hypoxic culture while disappearing under normoxic conditions. In contrast, miR-135b expression could be maintained under normoxic conditions. Thus, cobalt ion-driven hypoxia conditions may accelerate angiogenesis by changing exosomal miRNA expression. For integrin β1, exosomes significantly enhance the integrin β1 expression of endothelial cells upon cobalt ions stimulation, which might be beneficial for angiogenesis. Thus, an appropriate concentration of cobalt ions could induce a hypoxic microenvironment and facilitate macrophage secretion of exosomes, promoting angiogenesis. This also indicates the importance of controlling the release of cobalt ions from implants.

### 3.4. Calcium-Containing Biomaterials

As ionic messengers in cells, calcium ions play key roles in living systems, and are involved in many processes such as apoptosis, movement, signal transduction, gene transcription, and gene differentiation, etc. As essential inorganic components, 99% of calcium ions are stored in bone and present in the form of HA in combination with phosphate. It is important to maintain appropriate concentrations of intracellular and extracellular calcium ions during the formation of mature bone. Low Ca^2+^ concentrations (2–4 mM) in culture media improve osteoblast proliferation, medium concentrations (6–8 mM) enhance osteoblast differentiation and extracellular matrix mineralization, and high concentrations (>10 mM) are toxic [67]. Additionally, studies proved that calcium ions (3~5 mM) released from CaSO_4_-containing scaffolds enhance BMSCs migration, osteoblast gene expression, and new bone formation in a concentration-dependent manner by activating the PI3K–AKT pathway [68]. Another study showed that appropriate Ca^2+^ release from mesoporous silica xerogels could improve osteoblast proliferation and osteogenic differentiation by activating ERK1/2 signaling pathway [69].

**Figure 3:**
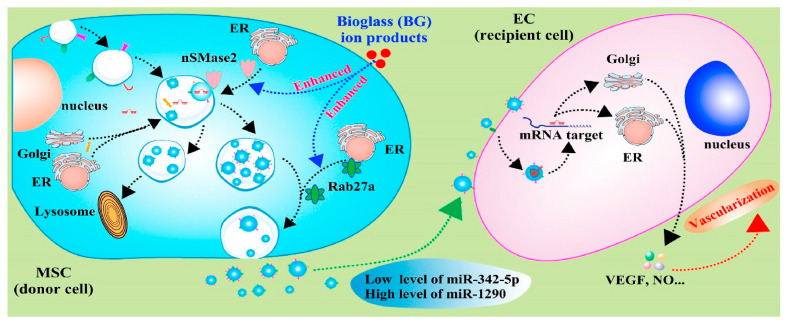
Schematic diagram indicates the mechanisms of bioglass ion products in regulating the production and function of exosome derived from MSCs, and the effects of secreted exosomes on angiogenesis of endothelial cells [72]. Reprinted with permission from KeAi Publishing.

With knowledge of exosomes in intercellular communication developing, the effects of calcium-containing biomaterials on exosomes production and their mechanisms are investigated. As the first bioactive glass introduced by Hench in 1971, 45S5 Bioglass^®^ is high in calcium content and shows good degradable capacity and bioactivity. In particular, 45S5 Bioglass^®^ is composed of 46.1 mol.% SiO_2_—26.9 mol.% CaO—24.4 mol.% Na_2_O—2.6 mol.% P_2_O_5_, which can form a strong bond with the surrounding bone. The high bioactivity of the material can be attributed to hydroxycarbonate apatite formation on its surface, which occurs due to glass dissolution. The dissolution products of bioglass (e.g., calcium and silica ions) have a positive effect on the proliferation and osteogenic differentiation of bone-forming-related cells, as well as the regulation of macrophage polarization [70,71]. Wu et al. [72] investigated the effects of ion products of 45S5 Bioglass on the exosome production of MSCs and found that ion products induced a two-fold increase in exosome production by up-regulating the expression of sphingomyelinase-2 (nSMase2) and Rab27a. The nSMases family and Rab family play key roles in vesicle formation and membrane traffic. nSMase2 facilitates ceramide formation and the budding of endosomal membranes, leading to enhanced intravesical formation [73]. Rab27a, located and expressed at CD63-positive multivesicular endosomes, regulates vesicle motility by docking into cell-specific compartments, playing a role on membrane-fusion process [74]. Thus, the ion produced by 45S5 Bioglass can affect the formation and release of exosomes by regulating SMase2 and Rab27a expression, which control nSMase and Rab GTPase pathways (Figure 3). In addition, the up-regulation of miR-1290 and the down-regulation of miR-342-5p in exosomes upon the stimulation of ion production have been proven to facilitate the vascularization capability of endothelial cells [72]. Studies have proven that miR-342-5p could repress VEGF-triggered Akt phosphorylation and decrease endoglin expression, which suppress the proliferation of endothelial cells and vascularization [75,76]. Additionally, miR-1290-overexpressing exosomes could promote tumor angiogenesis by attenuating the inhibition of VEGFR2 phosphorylation by targeting SMEK1 [77]. Thus, the exosomes with increased miR-1290 expression and decreased miR-342-5p expression, mediated by ion production, have shown enhanced vascularization capability [72].

In addition, calcium ions also regulate the exosome secretion derived from bone-resorbing cells. Calcium phosphate (CaP), as a calcium-rich biomaterial, has good biocompatibility. When amorphous-phase CaP nanoparticles were cocultured with macrophages, it was observed that the particles could increase the number of exosomes more than twofold [78]. However, CaP particle treatment had no significant effect on the calcium concentration in exosomes [78]. CaP particles entered into cells through the endocytosis or phagocytosis pathways and released Ca^2+^ in the acidic microenvironment (e.g., late endosomes or lysosomes). Then, a Ca^2+^ increase resulted in the collapse of their membranes and the Ca^2+^ release into cytosol, which induced the increase in intracellular calcium concentration and exosome release. Thus, CaP particles have potential applications for improving the production efficiency of exosomes, while not affecting the Ca^2+^ concentration in exosomes. Additionally, calcium oxalate was found to alter the protein expression levels in the macrophage-derived exosomes, which were involved in the immune response, cell migration, transcription regulation, and calcium binding [79,80]. Further study found that these exosomes could enhance IL-8 production and the migration of immune cells [79,80]. All these findings indicate that calcium-enriched microenvironments not only increase the exosome production of macrophages, but also enhance the proinflammatory response via the exosomal pathway.

### 3.5. Strontium-Containing Biomaterials

As a nonessential element, approximately 98% of strontium ions are stored in human bone tissue, and have strong bone-seeking behavior. Presently, strontium is widely used in the form of strontium ranelate as a treatment for osteoporosis, especially for postmenopausal women. Thus, strontium is incorporated into various biomaterials to enhance bone regeneration, including bioactive glass, calcium phosphate, and metallic implants [81,82]. The incorporation of Sr^2+^ could enhance ALP activity, osteogenic gene expression in osteoblastic cells, and calcium nodule deposition [82]. Additionally, the addition of Sr^2+^ enhances the migration and tube formation of endothelial cells, as well as increasing the expression of VEGF and Angiopoietin-1 (Ang-1) [82]. Additionally, Sr^2+^-containing bioactive microspheres can enhance the transformation of macrophages toward to M2 phenotypes and secrete high levels of PDGF-BB, resulting in the improved angiogenic capacity of endothelial cells and early vascularization in vivo [83]. However, the underlying mechanisms of paracrine communication remain unclear. Liu et al. [19] investigated the influence of the strontium-substituted calcium silicate on exosome-derived BMSCs and found that, after strontium stimulation, exosomes showed superior pro-angiogenic ability. This effect might be attributed to the increased level of miR-146a and the inhibition of Smad4 and NF2 proteins in exosomes. Studies proved that miR-146a is involved in the angiogenic process [84,85]. In addition, it was proposed that Smad4 and NF2 were potential targets of miR-146a. As a member of the Smad family, Smad4 serves as a vital regulator of TGF-β signaling pathways and suppresses tumor progression by inhibiting angiogenesis [86,87]. Additionally, as a component of the Hippo signaling pathway, the knockdown of NF2 can improve the angiogenesis of endothelial cells [88]. Thus, exosomes containing increased miR-146a, derived from BMSCs upon strontium-incorporation stimulation, could enhance angiogenesis by inhibiting the expression of Smad4 and NF2. This was also proven by the knockdown of miR-146a, accompanied by the increased protein expressions of Smad4 and NF2 in a report by Liu et al. [19]. Therefore, exosomes under the stimulation of strontium-incorporated biomaterials play a key role in vascularization and the following bone regeneration. This observation provides new insights into the underlying mechanisms of strontium-incorporated biomaterials in bone regeneration.

### 3.6. Lithium-Containing Biomaterials

Lithium ions have long been widely used to treat psychiatric disorders and bipolar disease. Due to its association with hyperparathyroidism, lithium has attracted great interest in bone tissue treatment. Zamani et al. [89] found that 75 patients treated with lithium carbonate had significantly greater bone density compared with normal patients, indicating the potential of lithium therapy for preserving or enhancing bone mass. Another study has proved that Li^+^ addition can enhance the proliferation of BMSCs by glycogen synthase-3β (GSK-3β) inhibition, and the β-catenin/Wnt activation that follows [90]. GSK-3β is a multifunctional protein kinase involved in diverse cellular metabolic processes such as cell proliferation, migration, and cell cycles. Inhibition of GSK-3β increased BMSCs migration through enhancing β-catenin expression, which was followed by the activation of Wnt-responsive genes [91]. Moreover, Li^+^ inhibits GSK-3, activating HIF-1 that induces vasculogenesis [92,93]. Thus, Li^+^ is a new additive for incorporation into bone substitutes due to its stimulating effects on vasculogenesis and bone formation. Since high glucose inhibits the migration of BMSCs through the activation of GSK-3β, Chen et al. [94] prepared Li^+^-containing bioactive glasses and found that Li^+^ addition could reverse the suppression of migration, proliferation, and osteogenic differentiation of BMSCs, which is induced by high glucose, by activating the β-catenin/Tcf7/Ccn4 signaling pathway. Moreover, the Li^+^ released from bioactive glasses has been proven to facilitate the proliferation and tubules formation of endothelial cells by activating the canonical Wnt/β-catenin pathway and increasing the expressions of proangiogenic cytokines including insulin-like growth factor 1 (IGF-1) and TGFβ [95].

**Figure 4:**
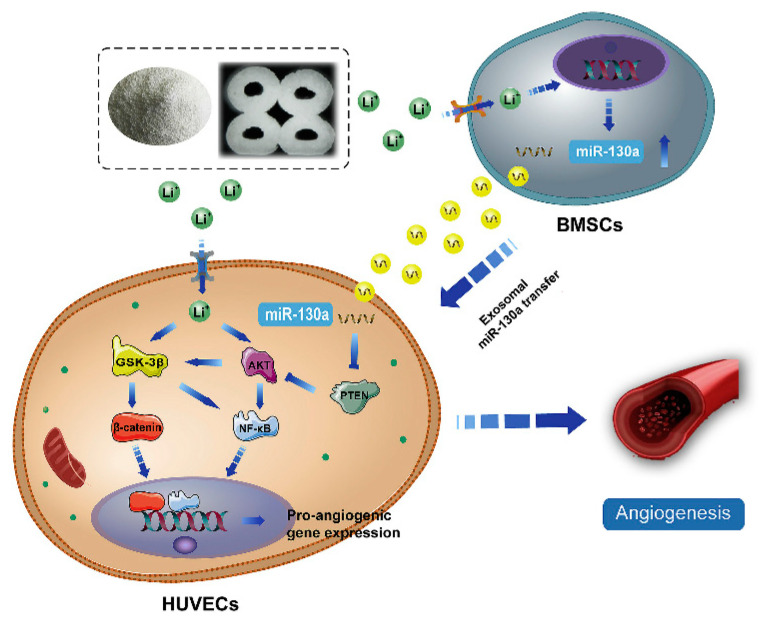
The potential mechanism of exosomes derived BMSCs upon stimulation of Li^+^-containing biomaterials in promoting angiogenesis of endothelial cells [96]. Reprinted with permission from Elsevier.

Despite advances in the exploration of the roles of Li^+^ in bone regeneration, the underlying mechanisms between intercellular communications remain unclear. During the bone healing process, the communication between BMSCs and endothelial cells is very important for vascularized bone regeneration. Liu et al. [96] found that Li-containing bioactive glass could promote the angiogenic capacity of endothelial cells by enhancing the expressions of miR-130a in exosomes derived from BMSCs and subsequently down-regulating the PTEN protein and activating the AKT signaling pathway (Figure 4). miR-130a plays a vital role in angiogenesis. First, miR-130a can maintain normal autophagy behavior and improve the survival of endothelial cells by the regulation of Beclin1 and Bcl-2 expression [97]. Second, miR-130a has been proven to enhance angiogenesis and tumor growth through targeting Runx3 [98]. More importantly, miR-130a has been proven to target the PTEN protein, and can thus prevent cerebral damage by activating the PI3K/AKT signaling pathway through the inhibition of PTEN [99]. Thus, exosomal miR-130a upon stimulation by Li^+^-containing bioactive glass is beneficial for communication between BMSCs and endothelial cells through paracrine secretion. Subsequently, it enhances angiogenesis by the activation of the PTEN/AKT signaling pathway, which can up-regulate HIF-1α expression and VEGF secretion [96].

## 4. Conclusions and Future Directions

During skeletal development and remodeling, exosomes derived from cells including immune cells and bone-forming-related cells, play crucial roles in regulating cell-to-cell communication and followed angiogenesis and osteogenesis. Moreover, metallic ions, either alone or incorporated with bone substitutes, have been proven to affect the amount or cargoes of exosomes, subsequent modification of cell adhesion, proliferation, and differentiation as well as bone regeneration (Table 3). Although limited studies have investigated the detailed mechanisms of the effects of metallic ions on angiogenesis and osteogenesis through their influence on exosome secretion, there is no doubt that metallic ions-mediated paracrine secretion-based methods present promising strategies for determining the angiogenic and osteogenic behavior of bone forming-related cells and the subsequent in vivo fate of bone implants. Future biomaterial designs should focus on modifying direct cell-material interactions as well as on generating a favorable microenvironment for enhancing cell behavior through paracrine communication. However, the underlying mechanism of exosome-mediated bone regeneration upon metallic ions stimulation remains unclear and require further study.

For an investigation of the effect of metallic ions on exosome secretion, the following issues must be resolved: First, the number of exosomes obtained in cell culture mediums is always not sufficient. Two possible approaches are in use at present: one is to use commercial isolation kits to increase the rate of recovery from the culture medium; the other is to stimulate the secretion of cell-derived exosomes as much as possible. Thus, the modification of bone substitutes by the incorporation of metallic ions may be a promising method for the creation of favorable microenvironments that can enhance the amount and biofunction of cell-secreted exosomes. Second, since our understanding of the underlying mechanism of the effect of (implanted) metallic ion-mediated exosomes on bone regeneration is still in its infancy, more efforts should be devoted into investigating the mechanism by how chemical signals derived from biomaterials affect exosomes, and how these exosomes affect the subsequent immune reaction, angiogenesis, and osteogenesis. To achieve this, the types and release kinetics of the metallic ion-incorporated bone substitutes should be further investigated so that we can work toward fabricating tailor-made biomaterials that generate an ideal microenvironment, enhancing exosome biofunctions and bone regeneration. Moreover, investigations into the roles of metallic ion-incorporated bone substitutes in relation to exosome secretion are currently focused on bioglasses and other bioceramics. Metallic alloy- and polymer-based bone substitutes should also be taken into consideration in future studies. Moreover, since exosomes play an important role in cell-to-cell communications, the effects of metallic ion-mediated exosomes on the interactions among different cells should be further studied. We do not currently fully understand how metallic ions regulate cell response by paracrine secretion, or how these responses affect the subsequent bone formation; however, through careful research design, it will be possible to investigate the role of exosomes in inflammation, angiogenesis, and osteogenesis. Such research advances would enrich our understanding of the biological mechanism of metallic ion-mediated bone regeneration, and would be beneficial for the design of metallic ion-incorporated bone substitutes. Similarly, in addition to chemical composition, the surface structure and mechanical signals derived from biomaterials should be explored when investigating the stimulation effects on exosomes secretions. Finally, due to their low immune rejection and high therapeutic effects, metallic ion-stimulated functional exosomes can serve as potential drugs for various clinical diseases.

## Figures and Tables

**Table 2:** Exosomes derived from different macrophages and their functions in bone remodeling.

Sources	Markers	Cargoes	Function	Ref.
M1-like macrophages-derived exosomes	CD63, Hsp70	Undetected	Accelerate bone loss in postmenopausal osteoporosis via enhancing miR-98 expression and subsequent downregulation of DUSP1 and activation of JNK signaling pathway in osteoblasts;	[38]
	CD9, Tsg101	Enriched miR-155	Restrain MSCs osteogenic differentiation by inhibiting BMP2 signaling pathway;	[15]
	CD81, CD63, CD9, Alix	Undetected	Support the proliferation, osteogenic and adipogenic differentiation of BMSCs, rather than exosomes derived from M2-like macrophages	[36]
M2-like macrophages-derived exosomes	CD63, CD81	miRNA-5106 overexpression	Enhance the osteogenic differentiation of BMSCs via inhibiting the expression of SIK2 and SIK3 genes, facilitate bone fracture healing;	[37]
	CD9, TSG101	Enrich miR-378a	Increase osteogenic differentiation of MSCs via enhancing BMP signaling pathway;	[15]
	CD63, CD81	Undetected	Inhibit adipogenesis and enhance osteogenesis of BMSCs via miR-690/IRS-1/TAZ axis	[14]

**Table 3:** Exosomes derived from different cells upon metallic ions stimulation and their effects on bone repair.

Sources	Metallic ion Stimulation	Cargoes	Function	Ref.
Macrophages-derived exosomes	Mg^2+^	miR-381	Enhance osteogenic differentiation of BMSCs by promoting M2 polarization and decreasing miR-381 in exosomes via autophagy;	[49]
	Cu^2^^+^	Undetected	Enhance angiogenic capacity of endothelial cells probably by upregulating the pro-angiogenic RNAs and downregulate anti-angiogenic RNAs in exosomes.	[58]
	Co^2+^	Undetected	Enhance endothelial migration and angiogenesis via upregulating NO, VEGF and integrin β1 expression;	[63]
	Ca^2+^	Undetected	Increase the production of exosomes that are not contaminated by Ca^2+^; enhance inflammation response by increasing IL-8 and IL-1β production	[78,79,80]
MSCs-derived exosomes	Ca^2+^	miR-1290 miR-342-5p	Induce a two-fold increase in exosome production via enhancing the expression of nSMase2 and Rab27a; facilitate vascularization capability of endothelial cells via miR-1290 upregulation and miR-342-5p downregulation in exosomes;	[72]
	Sr^2+^	miR-146a	Enhance angiogenesis via suppressing the expression of Smad4 and NF2 through increased miR-146a in exosomes;	[19]
	Li^+^	miR-130a	Enhance the angiogenic capacity of endothelial cells via down-regulating PTEN protein and activating AKT signaling pathway through enhancing the expressions of miR-130a in exosomes	[96]

## Data Availability

Not applicable.
